# Exosomal miR-423-5p Derived from Cerebrospinal Fluid Pulsation Stress-Stimulated Osteoblasts Improves Angiogenesis of Endothelial Cells via DUSP8/ERK1/2 Signaling Pathway

**DOI:** 10.1155/2024/5512423

**Published:** 2024-05-11

**Authors:** Hailong Li, Yiqun He, Xujun Chen, Aolei Yang, Feizhou Lyu, Youhai Dong

**Affiliations:** ^1^Department of Orthopedics, Shanghai Fifth People's Hospital, Fudan University, Shanghai, China; ^2^Department of Orthopedics, Huashan Hospital, Fudan University, Shanghai, China

## Abstract

Exosomes secreted from osteoblasts (OBs) can regulate the angiogenesis of endothelial cells (ECs); however, whether cerebrospinal fluid pulsation (CSFP) stress, a special mechanical stimulation, can influence the cell's communication in the context of angiogenesis remains unknown. In this study, the effect of exosomes derived from CSFP stress-stimulated OBs on facilitating the angiogenesis of ECs was investigated. First, OBs were cultured in a CSFP bioreactor, and exosomes derived from OBs were isolated and identified. Cell Counting Kit 8 assay, transwell migration assay, wound healing migration assay, and tube formation assay were conducted to assess the effects of CSFP stress-stimulated OBs-derived exosomes (CSFP-Exos) on the angiogenesis of ECs. Then high-throughput RNA sequencing was used to determine the miRNA profiles of Non-CSFP stress-stimulated OBs-derived exosomes (NCSFP-Exos) and CSFP-Exos, and the luciferase reporter gene assay was performed to confirm the binging of miR-423-5p to DUSP8. In addition, the Matrigel plug assay was performed to explore whether exosomal miR-423-5p has the same effects *in vivo*. Our results suggested that CSFP-Exos can promote the angiogenesis of ECs, and miR-423-5p was enriched in CSFP-Exos. Moreover, miR-423-5p could promote the effect of angiogenesis via directly targeting dual-specificity phosphatase 8 (DUSP8), which inhibited the ERK1/2 signaling pathway. In conclusion, exosomal miR-423-5p derived from CSFP stress-stimulated OBs could promote the angiogenesis of ECs by the DUSP8/ERK1/2 signaling pathway.

## 1. Introduction

Bone tissue engineering has great potential to repair the vertebral lamina defect. During artificial laminae reconstruction, blood vessel growth and osteogenesis are tightly interconnected biological processes [1, 2]. The growth of blood vessels is a prerequisite for bone formation, providing oxygen, nutrients, and cells [3, 4], and many studies have focused on exploring the effect of mechanical factors on facilitating angiogenesis. Studies showed that pulsatile mechanical stimulation could not only promote the differentiation of mesenchymal stem cells (MSCs) into osteoblasts (OBs) and endothelial cells (ECs) [5, 6] but also induced OBs to secrete angiogenic factors and related chemokines and promote the proliferation and migration of ECs [7]. Previously, we reported that cerebrospinal fluid pulsation (CSFP) stress could promote the vascularization [8], osteogenesis [9], and remodeling of tissue-engineered laminae (TEL) [10], and in another study, we showed that exosomes derived from OBs could effectively promote the proliferation, migration, and tube formation of ECs [11]. However, whether CSFP stress can regulate angiogenesis through exosomes derived from OBs is still unknown.

Exosomes, extracellular vesicles ranging from 30 to 150 nm in size, act as key mediators of intercellular communication by delivering proteins, mRNAs, and miRNAs to recipient cells [12, 13]. Exosomes can be considered as a kind of biological material in the field of regeneration medicine for its excellent cellular internalization and biocompatibility. Numerous studies have indicated that exosomes derived from different cell types can facilitate the angiogenesis of ECs, in which exosomal miRNAs play a crucial role [14–16]. Based on previous studies, we aimed to investigate whether exosomal miRNAs derived from CSFP stress-stimulated OBs can promote the angiogenesis of ECs.

In this study, we identified that CSFP stress can stimulate the secretion of OBs-derived exosomal miR-423-5p, and exosomal miR-423-5p can be transferred to ECs, thereby promoting angiogenesis by targeting DUSP8. In addition, we demonstrated that exosomal miR-423-5p mediated angiogenesis by regulating the ERK1/2 signaling pathway. To the best of our knowledge, our study is the first time to show CSFP stress can stimulate the secretion of OBs-derived exosomal miR-423-5p which promotes angiogenesis and elucidate the underlying mechanism.

## 2. Materials and Methods

### 2.1. Cell Culture

MC3T3-E1 cells (SCSP-5218) and ECs bEnd.3 (TCM40) were purchased from the Cell Bank at the Chinese Academy of Science, Shanghai, China. MC3T3-E1 cells were cultured in Minimum Essential Medium *α* (MEM *α*, Hyclone, USA, Cat SH30265.01) with 10% exosomes depleted fetal bovine serum (FBS, Gibco, Cat 10437-028) and 100 U/mL penicillin-streptomycin (Gibco, USA, Cat 15140122). For osteogenesis, MC3T3-E1 cells were incubated in the osteogenic induction medium with *β*-glycerophosphoric acid (10 mM) (Aladdin, China, Cat C2226462) and L-ascorbic acid (50 mg/L) (Sigma, USA, Cat SLBX2310). bEnd.3 cells were cultured in Dulbecco's Modified Eagle Medium (DMEM, Hyclone, USA, Cat SH30022.01) with 10% FBS and 100 U/mL penicillin-streptomycin.

### 2.2. Exosomes Isolation, Purification, and Characterization

According to the manufacturer's protocol for the Exo quick reagent (Invitrogen, USA, Cat 4478359), exosomes were isolated with some adjustments. Briefly, 15 mL MC3T3-E1 cell culture medium was centrifuged at 3,000 x *g* for 10 min to eliminate cells and filtered with a 0.22 *µ*m filter to discard dead cells and debris. The supernatant was further ultracentrifuged at 4,000 x *g* for 60 min at 4°C using 100 kDa ultrafiltration filter devices (Millipore, USA, Cat UFC9100). After adding an equal volume of the Total Exosome Isolation and mixing well, the mixture was incubated at 4°C overnight. Then, the mixture was centrifuged at 12,000 x *g* for 60 min at 4°C, and the supernatant was discarded. Exosomes were collected from the final pellet and then resuspended by 1x phosphate-buffered saline (PBS, Servicebio, China, Cat G4202-100ML) and stored at −80°C until use. The exosomes were identified by western bolting, nanoparticle tracking analysis (NTA), and transmission electron microscopy (TEM). The BCA Protein Assay Kit (Thermo, USA, Cat 23225) was used for the quantitative analysis of exosomes.

### 2.3. Exosome Internalization

PKH-67 green fluorescent dye (Sigma, USA, Cat MKCK8884) was used for labeled exosomes derived from OBs according to the manufacturer's protocol. The labeled exosomes were incubated with bEnd.3 cells for 12 hr, and the uptake was quantified by immunofluorescence.

### 2.4. Cell Transfection

MiR-423-5p mimic, miR-423-5p NC, miR-423-5p inhibitor, and Cy3-labeled-miR-423-5p mimic were synthesized by Ribo-Bio. Cell transfection was performed using Lipofectamine 2000 (Invitrogen, USA, Cat 11668019) following the manufacturer's instructions.

### 2.5. Tube Formation Assay

bEnd.3 cells (2 × 10^4^ cells per well) were seeded onto Matrigel-coated (50 *µ*L/well) 96-well plates. bEnd.3 cells were incubated with PBS, NCSFP-Exos, CSFP-Exos, or exosomes previously derived from OBs transfected with miR-423-5p mimic, miR-423-5p NC, and miR-423-5p inhibitor. bEnd.3 cells were treated with 20 *μ*M PD98059 (MedChemExpress, USA, Cat HY-12028) and then incubated with exosomes previously derived from OBs transfected with miR-423-5p mimic.

### 2.6. Wound Healing Assay

bEnd.3 cells were seeded in six-well plates and cultured overnight. The separate wounds were scratched by a 200 *μ*L pipet tip, and 1x PBS was utilized to wash cells. Subsequently, cells were cultured with a serum-free medium. The images of migrated cells were photographed using phase-contrast microscopy at 0 and 24 hr.

### 2.7. Cell Migration Assay

Cell migration was detected using a 24-well transwell plate (8 *μ*m polycarbonate filter, Corning, USA, Cat 3422). Cells were seeded in the plate at a cell density of 3 × 10^5^ cells/mL into the upper chamber with 100 *μ*L per chamber, while 600 *μ*L of DMEM containing 10% FBS was added into the lower chamber. After incubation for 24 hr, cells on the upper surfaces of upper chambers were removed with cotton-tipped swabs, and cells migrated to the lower surface were fixed in formaldehyde for 20 min and then stained with crystal violet staining solution (Beyotime, China, Cat Co121-100ml) and counted.

### 2.8. Cell Proliferation Assay

The Cell Counting Kit-8 assay (CCK-8, Dojindo, Japan, Cat CK04) was applied to assess bEnd.3 cells proliferation. bEnd.3 cells were seeded in a 96-well plate with 5,000 cells/well with three replicates. After 0, 24, 48, and 72 hr culture, cell proliferation was detected by Tecan Infinity 200 Pro-multi-well plate reader (Switzerland) at 450 nm.

### 2.9. Western Blotting

bEnd.3 cells were extracted, and protein concentration was quantified by BCA Protein Assay Kit (Thermo, USA, Cat 23225). About 20 *μ*g protein was loaded and separated by 10% SDS–PAGE gel and transblotted onto PVDF Membranes (Millipore, USA, Cat ZY101123). After being blocked with 5% nonfat dried milk, the membranes were incubated with primary antibodies for *β*-actin (CST, USA, Cat 4967S), CD63 (ABclonal, China, Cat A19023), CD81 (ABclonal, China, Cat A22528), DUSP8 (ABclonal, China, Cat A17990), ERK1/2 (CST, USA, Cat 4695S), pERK1/2 (CST, USA, Cat 4376S) at 4°C overnight. TBST was used to wash membranes, and then membranes were incubated with goat anti-mouse IgG-horseradish (CST, USA, Cat 7076S) or goat anti-rabbit peroxidase secondary antibody (CST, USA, Cat 7054S). Finally, bands were detected by an ECL Detection kit (Share-bio, China, Cat PK10003) and imaged.

### 2.10. RNA Extraction and Reverse-Transcription PCR

Total RNAs were isolated using TRIzol reagent (Invitrogen, USA, Cat 15596026). MiRcute Plus miRNA First-Strand cDNA Kit (TIANGEN, China, Cat KR211-02) and MiRcute Plus miRNA qPCR Kit (SYBR Green) (TIANGEN, China, Cat FP411-01) were used for qualification of miRNA, and U6 was used as the internal control. qPCR was performed on the Applied Biosystems 7500 Real-Time PCR Detection System (Applied Biosystems, CA, USA). The following primer sequences are listed in Table 1.

### 2.11. Luciferase Reporter Gene Assay

To evaluate the direct binding between miR-423-5p and the 3′UTR of DUSP8, HER293T cells in a 48-well were co-transfected with 50 nM miR-423-5p mimic or NC duplex and 1 *μ*g of dual-luciferase reporter vector. Forty-eight hours after transfection, the luciferase assay was measured according to the manufacturer's instructions.

### 2.12. Matrigel Plug Assay

The protocol was approved by the Committee on the Ethics of Animal Experiments of East China Normal University (No. m20210910). 4 × 10^6^ bEnd.3 cells were incubated with exosomes derived from miR-423-5p NC/miR-423-5p mimic/miR-423-5p inhibitor pretreated OBs and 4 × 10^6^ bEnd.3 cells preprocessed with PD98059 were incubated with exosomes derived from miR-423-5p mimic pretreated OBs. The cell suspensions were mixed with 500 *μ*L of Matrigel (BD, USA, Cat 354234) at a ratio of 1 : 1. First, the inhalation anesthetic isofluorane was used before animal experimentation. Then, we subcutaneously injected cell suspension into the upper back of the nude mice (female, 6-weeks old, BALB/c). The solution had a temperature of 4°C at the time of injection, and it solidified at 37°C. After 14 days, the nude mice were first euthanized by exposure to an increasing concentration of carbon dioxide; death was then confirmed by a physical process, and Matrigel plugs were collected, photographed, and performed for CD31 (1 : 100; Servicebio, China, Cat GB11063-2-100) immunofluorescence staining and DUSP8 (1 : 100; ABclonal, China, Cat A17990), pERK (1 : 100; Servicebio, China, Cat GB11507-100), and VEGF (1 : 100; Servicebio, China, Cat GB11034B-100) immunohistochemistry (IHC).

### 2.13. Statistical Analysis

Statistical analysis was conducted using GraphPad Prism version 9 for Mac (9.0a, USA). All data are presented as the means ± SD. Student's *t*-test and one-way ANOVA were conducted to compare the differences between two groups or more than two groups.  ^*∗*^*P* < 0.05 was considered to be statistically significant.

## 3. Results

### 3.1. Isolation, Characterization, and Internalization of OBs-Derived Exosomes

**Figure 1:**
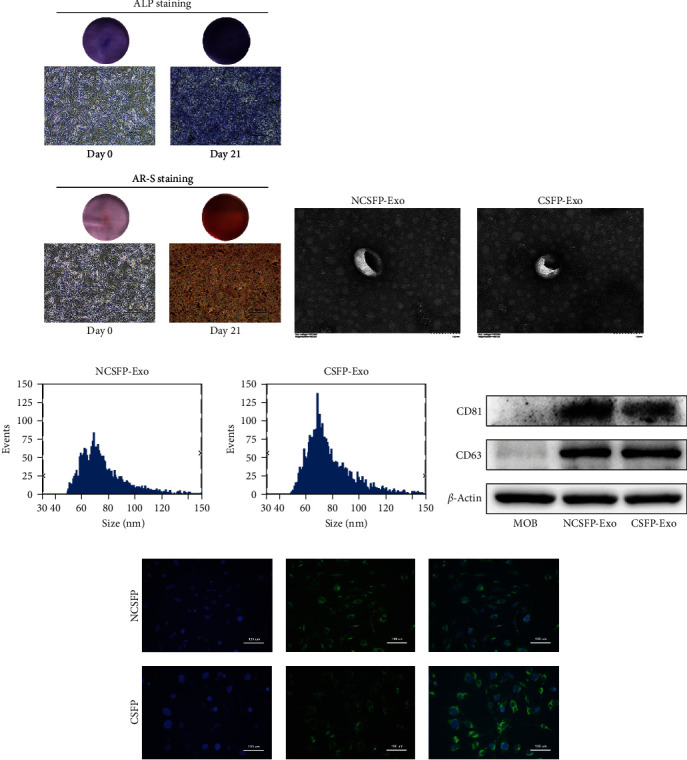
(a) ALP staining and Alizarin red staining at 21 days; (b) TEM images of NCSFP-Exos and CSFP-Exos; (c) particles size distribution of NCSFP-Exos and CSFP-Exos; (d) exosome surface markers CD81 and CD63 were detected by western blotting; (e) ECs incubated with NCSFP-Exos and CSFP-Exos labeled with PKH-67 (green), nuclei were stained with DAPI (blue).

As shown by the ALP staining and Alizarin Red S staining (Figure 1(a)), MC3T3-E1 was cultured in an osteogenic induction medium for 21 days to get OBs and then cultured under the stimulation of CSFP for 7 days. Exosomes were isolated and purified as described above.

TEM, NTA, and western blotting were performed to identify OBs-derived exosomes. As TEM images show, OBs-derived exosomes have a similar cup or sphere-shaped morphology (Figure 1(b)). The NTA analysis showed that the size of the particles of exosomes predominantly ranged from 48.25 to 149.25 nm (Figure 1(c)). Western blotting confirmed the expression of exosome surface makers, including CD63 and CD81 (Figure 1(d)). All these results suggested that these nanoparticles were exosomes.

To demonstrate whether OBs-derived exosomes can be taken up by ECs, ECs were cocultured with PKH67-labeled exosomes for 12 hr and then visualized with fluorescence microscopy. The results showed PKH67-labeled exosomes surrounded the nuclei, indicating ECs can uptake OBs-derived exosomes effectively (Figure 1(e)).

### 3.2. CSFP-Exos Promote Migration, Tube Formation, and Proliferation of ECs

**Figure 2:**
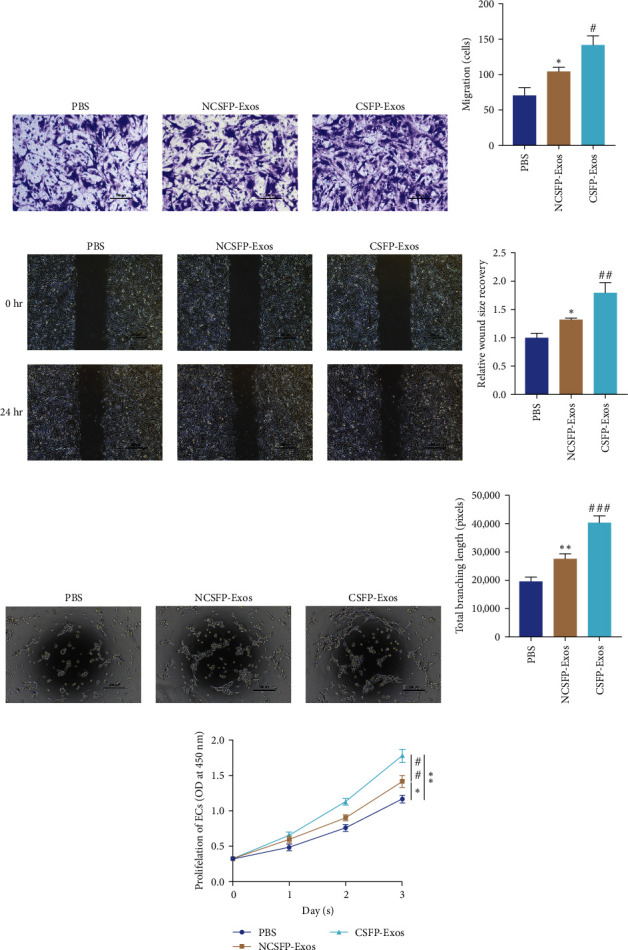
(a and b) NCSFP-Exos and CSFP-Exos promoted ECs migration measured by transwell (8 *μ*m) assay; (c and d) NCSFP-Exos and CSFP-Exos promoted ECs migration measured by wound healing assay; (e and f) tube formation assay of ECs cocultured with NCSFP-Exos and CSFP-Exos; (g) the proliferation of ECs in groups. Data are represented as mean ± SD; *n* = 3.  ^*∗*^*P*  < 0.05 vs. the PBS.  ^*∗∗*^*P* < 0.01 vs. the PBS, ^#^*P* < 0.05 vs. the NCSFP-Exos, ^##^*P* < 0.01 vs. the NCSFP-Exos, ^###^*P* < 0.001 vs. the NCSFP-Exos.

After coculturing ECs with CSPF-Exos, NCSPF-Exos, and an equal volume of PBS, ECs incubated with CSPF-Exos exhibited increased cell migration, which is detected by Transwell chamber migration assay and wound healing assay. The results showed both NCSFP-Exos and CSFP-Exos facilitated the migration and wound healing of ECs, and this facilitation of CSFP-Exos was greater than NCSFP-Exos (Figure 2(a)−2(d)). Tube formation assay was used to evaluate the capillary network formation of ECs. In the CSFP-Exos treated group, the cells demonstrated the strongest tube-forming ability (Figures 2(e) and 2(f)). Compared with the NCSFP-Exos treated group, the CCK8 assay suggested CSFP-Exos possessed a better effect on the proliferative abilities of ECs (Figure 2(g)). All the above evidence indicated that exosomes derived from OBs can promote the angiogenic ability of ECs. However, CSFP-Exos demonstrated a more excellent promotion.

### 3.3. Differential miRNA Expression Profile in CSFP Stress-Induced OBs-Derived Exosomes

**Figure 3:**
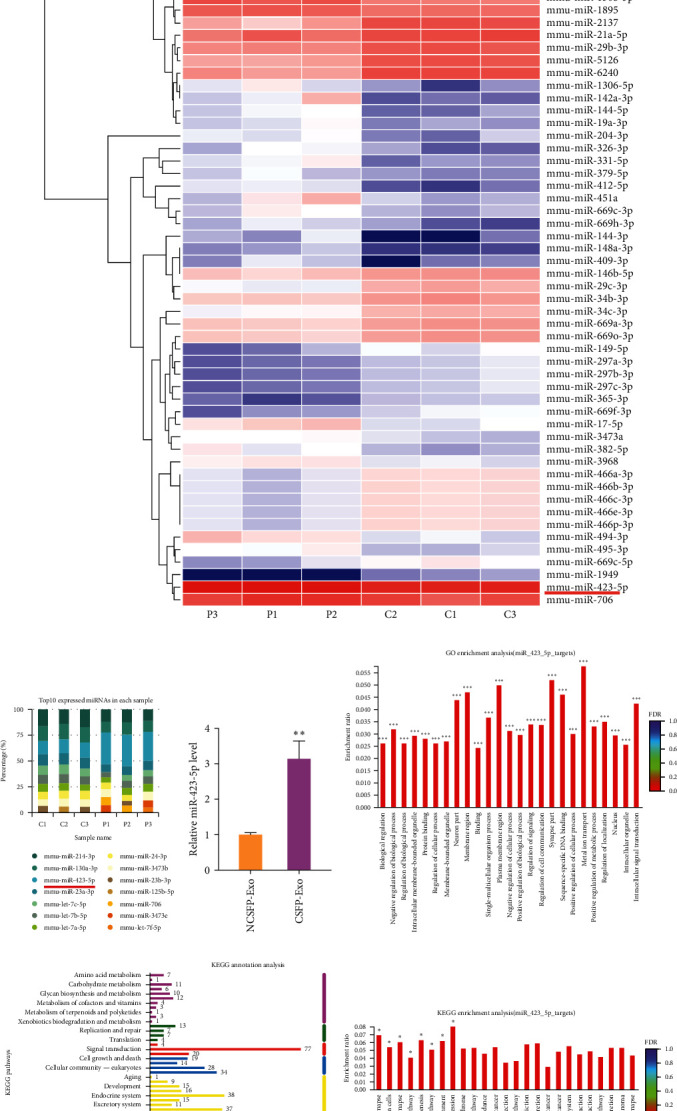
(a) Heatmap showing *Z*-score of miRNAs from NCSFP-Exos and CSFP-Exos. MiR-423-5p is one of the exosomal miRNAs with obviously greater abundance in CSFP-Exos compared with NCSFP-Exos; (b) the expression of miR-423-5p was significantly different in NCSFP-Exos and CSFP-Exos; (c) expression level of miR-423-5 p in CSFP-Exos was higher than in NCSFP-Exos; (d) gene ontology (GO) enrichment analysis; (e) Kyoto encyclopedia of genes and genomes (KEGG) annotation analysis; (f) KEGG enrichment analysis.  ^*∗*^*P* < 0.05 vs. the NCSFP-Exos,  ^*∗∗*^*P* < 0.01 vs. the NCSFP-Exos,  ^*∗∗∗*^*P* < 0.001 vs. the NCSFP-Exos.

**Figure 4:**
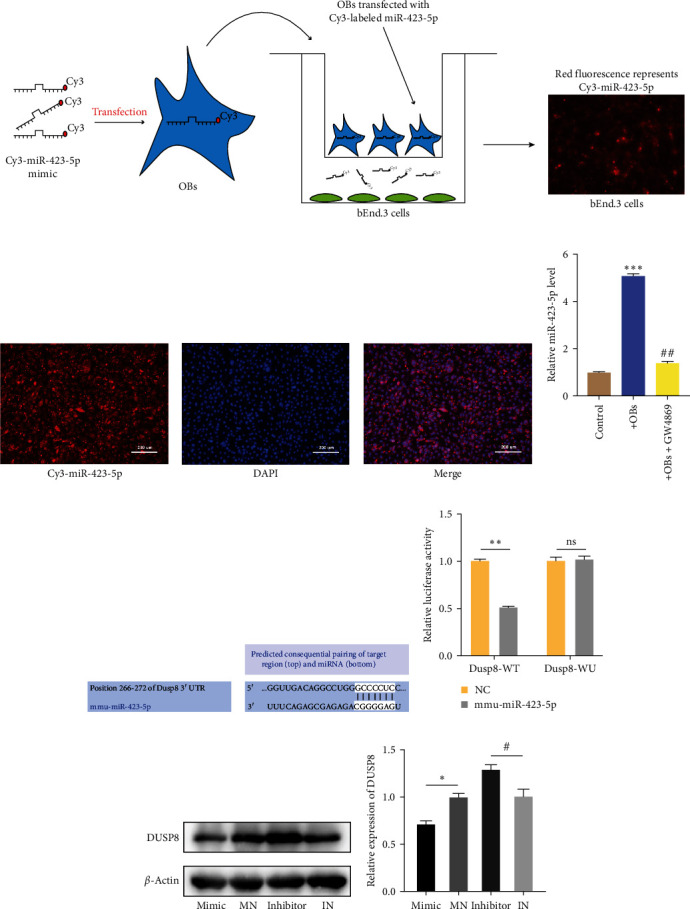
(a) bEnd.3 cells cocultured with OBs transfected with Cy3-labeled miR-423-5p mimic (red) in a transwell (0.4 *μ*m) plate; (b) representative images of OBs transfected with Cy3-miR-423-5p mimic; (c) the effects of GW4869 on exosome-dependent miRNA delivery from OBs into bEnd.3 cells; (d) the binging site for miRNA in DUSP8 mRNA; (e) a luciferase report assay; (f and g) western blotting. Data are represented as mean ± SD; *n* = 3.  ^*∗*^*P* < 0.05 vs. the Mimic,  ^*∗∗*^*P* < 0.01 vs. the Dusp8-WT NC,  ^*∗∗∗*^*P* < 0.001 vs. the Control, ^#^*P* < 0.05 vs. the Inhibitor, ^##^*P* < 0.01 vs. the +OBs, ^ns^*P* > 0.05 vs. Dusp8-WU NC.

Studies support that exosomal miRNAs are important regulators of biological activities [17, 18]. To identify the mechanism by which CSFP-Exos mediate ECs migration, tube formation, and proliferation, miRNA-seq was performed to profile the abundance difference of miRNAs between CSFP-Exos and NCSFP-Exos, and NCSFP-Exos was profiled as a control group (Figure 3(a)). Among these top 10 expressed miRNAs in samples, miR-423-5p was enriched in CSFP-Exos compared to NCSFP-Exos (Figure 3(b)). The expression of miR-423-5p was measured by qRT-PCR, which validated the results of miRNA-seq (Figure 3(c)). Among the Kyoto Encyclopedia of Genes and Genomes (KEGG) pathways, “Ras signaling pathway,” “Wnt signaling pathway,” “Relaxin signaling pathway,” “MAPK signaling pathway,” and “cGMP-PKG signaling pathway,” have been reported to be related to angiogenesis. Gene ontology (GO) enrichment analysis and KEGG enrichment analysis help us to know the gene ontology classification and to further explore the possible pathways in which targets were involved (Figure 4(d)–4(e)).

### 3.4. Exosomal miR-423-5p Can Be Secreted from CSFP Stress-Induced OBs and DUSP8 Is a miR-423-5p Target Gene

Results showed that CSFP-Exos could promote the angiogenesis of ECs, and miR-423-5p was enriched in CSFP-Exos, compared with NCSFP-Exos. Thus, we focused on miR-423-5p in the subsequent experiments.

After CSFP stress stimulation, miR-423-5p was packaged into exosomes and then transferred to bEnd.3 cells, yet it was unclear whether miR-423-5p was mainly transferred by exosomes. To examine the delivery method of OBs-derived exosomal miR-423-5p, Cy3-miR-423-5p mimic (red fluorescence) were used to transfer into OBs, and then coculture with ECs in a transwell plate (Figure 4(a)). The appearance of red fluorescence in ECs demonstrated that Cy3-miR-423-5p mimic was delivered from the OBs in the upper chamber to the recipient bEnd.3 seeded in the lower chamber (Figure 4(a)). The appearance of red fluorescence in OBs showed that the Cy3-miR-423-5p mimic (red fluorescence) was successfully transferred into OBs (Figure 4(b)). After coculture of OBs with bEnd.3 cells, a several-fold increase in OBs-derived miR-423-5p expression was detected by qRT-PCR. Additionally, the pretreatment of extracellular vesicle inhibitor GW4869 blocked exosome production of OBs; thus, the miR-423-5p expression in bEnd.3 was similar to coculture without OBs (Figure 4(c)).

Overall, our results suggested that miR-423-5p was mostly secreted in the form of exosomes.

To explore by which mechanism exosomal miR-423-5p-regulated angiogenesis, we used TargetScan and miRDB databases to identify miR-423-5p target genes. After detailed sequence analysis, we found that 3′UTR of DUSP8 exhibited a putative binding site of miR-423-5p (Figure 4(d)). Dual-luciferase reporter assay revealed that cotransfection of miR-423-5p mimic inhibited the activity of firefly luciferase reporter carrying the wild-type 3′UTR of DUSP8 (Figure 4(e)). In addition, we checked the DUSP8 level in transfected cells; treatment with miR-423-5p mimic significantly suppressed the expression of DUSP8 in bEnd.3, and cells transfected with miR-423-5p inhibitor exhibited a high level of DUSP8 (Figure 4(f)). The data indicated that exosomal miR-423-5p derived from OBs may promote angiogenesis by inhibiting DUSP8 in ECs.

### 3.5. Exosomal miR-423-5p Derived from OBs Promotes Angiogenesis of ECs by DUSP8/ERK1/2 Signaling Pathway In Vitro

**Figure 5:**
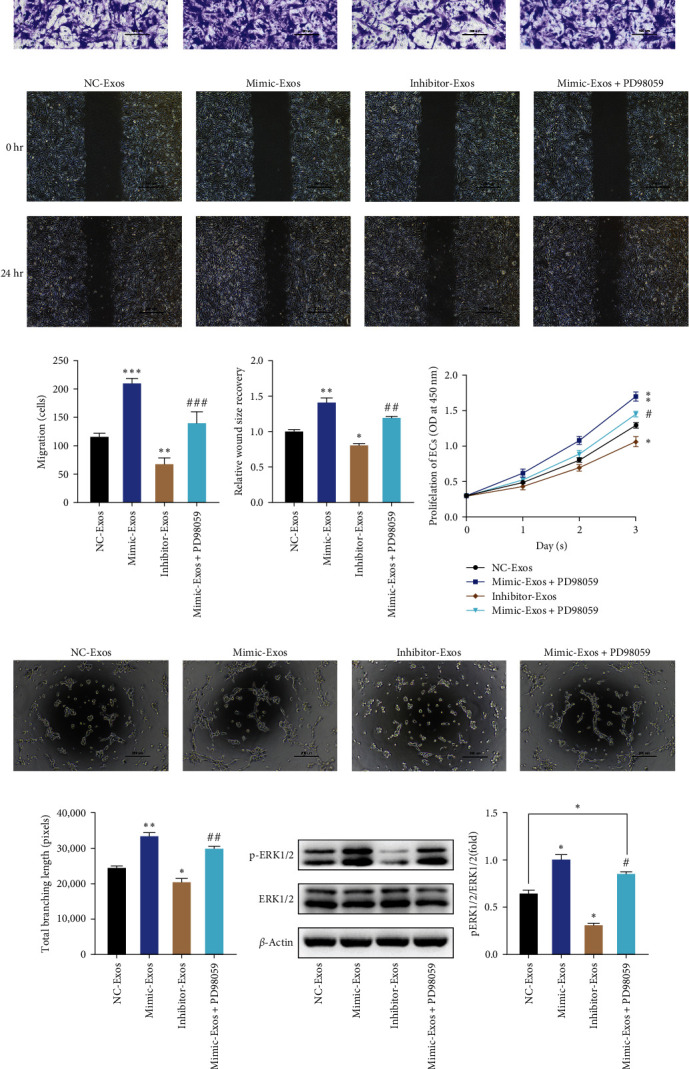
(a–d) Exosomal miR-423-5p promotes the migration of ECs, and ERK1/2 inhibitor weakens the effects; (e) the proliferation of ECs in groups; (f and g) tube formation of ECs in groups; (h and l) western blotting analysis of p-ERK1/2 and ERK1/2 in groups. All data were expressed as the mean ± standard deviation.  ^*∗*^*P* < 0.05 vs. the NC-Exos,  ^*∗∗*^*P* < 0.01 vs. the NC-Exos,  ^*∗∗∗*^*P* < 0.001 vs. the NC-Exos, ^#^*P* < 0.05 vs. the Mimic-Exos, ^##^*P* < 0.01 vs. the Mimic-Exos, ^###^*P* < 0.001 vs. the Mimic-Exos.

Transwell assay and wound healing assay were performed again to investigate the effect of exosomal miR-423-5p on the migration of ECs. Results indicated that exosomal miR-423-5p can distantly increase the migration of cells, and PD98059 weakened the facilitation but was still higher than the NC-Exos group (Figure 5(a)–5(d)). Besides, PD98059 withdrew the promotion in the proliferation of ECs compared with the Mimic-Exos group (Figure 5(e)). The tube formation assay showed PD98059 and also attenuated the effect of exosomal miR-423-5p in enhancing tube length and meshes number of ECs (Figures 5(f) and 5(g)). Importantly, western blotting indicated the expression level of phosphorylated ERK 1/2 (pERK1/2) in ECs was significantly decreased after ECs were cocultured with exosomal miR-423-5p derived from OBs which were pretreated with miR-423-5p inhibitor. The expression level of pERK1/2 in the Mimic-Exos group was higher than in the NC-Exos group, but the ERK1/2 inhibitor could still attenuate the expression level of pERK1/2 in the Mimic-Exos group. All the evidence demonstrated that exosomal miR-423-5p derived from OBs could increase migration, proliferation, and tube formation of ECs by DUSP8/ERK1/2 signaling pathway.

### 3.6. Exosomal miR-423-5p Promotes Angiogenesis of ECs by DUSP8/ERK1/2 Signaling Pathway In Vivo

**Figure 6:**
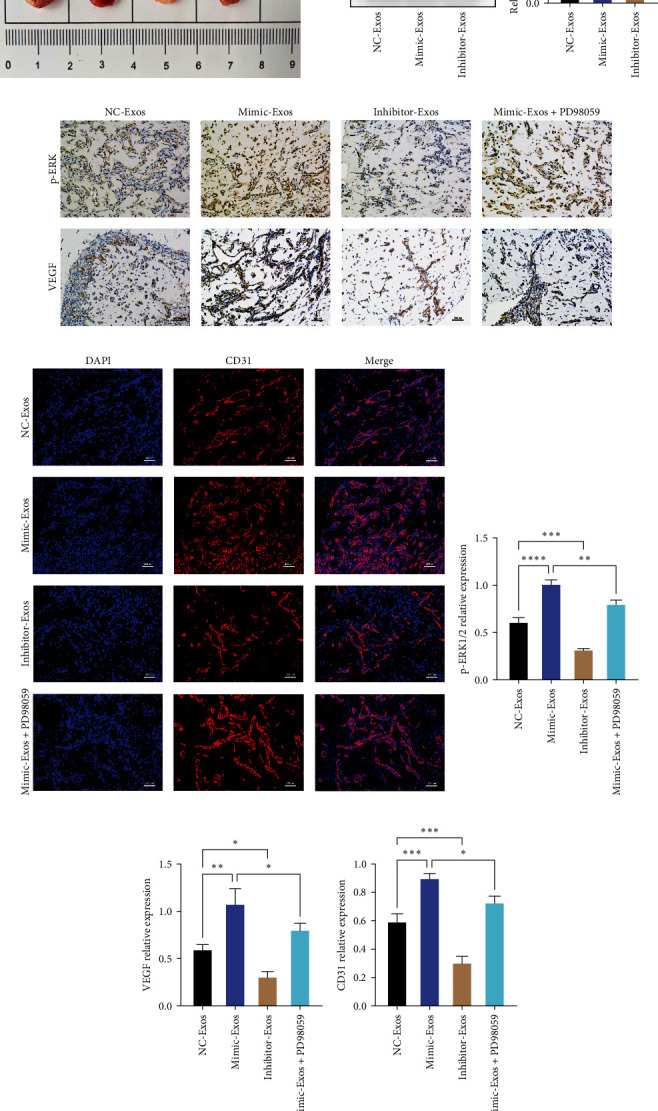
Exosomal miR-423-5p regulates angiogenesis in vivo: (a) image of Matrigel plugs after resection at 14 days; (b and c) western blot analysis of DUSP8 expression levels; (d, f, and g) immunohistochemical images analysis of p-ERK and VEGF-positive from Matrigel plugs; (e and h) images of CD31 detected by immunofluorescence staining in groups.  ^*∗*^*P* < 0.05 vs. the NC-Exos,  ^*∗∗*^*P* < 0.01 vs. the NC-Exos,  ^*∗∗∗*^*P* < 0.001 vs. the NC-Exos,  ^*∗∗∗∗*^*P* < 0.0001 vs. the NC-Exos.

The previous experiments showed that exosomal miR-423-5p derived from OBs could promote the angiogenesis of ECs by DUSP8/ERK1/2 signaling pathway in vitro. However, whether exosomal miR-423-5p has the same effect in vivo remains unknown. In this part, a Matrigel plug assay was performed to detect whether it can enhance angiogenesis in vivo. Compared to the Matrigel with exosomes of OBs-miR-423-5p NC, plug containing exosomes of OBs-miR-423-5p mimic indicated more efficient recruitment of ECs, and ERK1/2 signaling pathway inhibitor recedes this effect, suggesting that exosomal miR-423-5p efficiently increase angiogenesis in vivo (Figure 6(a)). The Mimic-Exos group showed a lower expression of DUSP8 than the NC-Exos group, and the Inhibitor-Exos group possessed the highest level (Figures 6(b) and 6(c)). IHC staining for pERK1/2 and VEGF showed the expression of pERK1/2 and VEGF in the Mimic-Exos group were higher than NC-Exos and Mimic-Exos+PD98059 group, and Mimic-Exos+PD98059 group exhibited a lower expression of pERK1/2 and VEGF than Mimic-Exos group (Figures 6(d), 6(f), and 6(g)). Also, immunofluorescence staining for CD31 was used to detect the extent of blood vessel formation.  Figure 6(e) shows that the blood vessels were abundant in the Mimic-Exos and Mimic-Exos+PD98059 groups; the abundance of blood vessels in the Mimic-Exos group was higher than Mimic-Exos+PD98059 group. These results suggested that exosomal miR-423-5p can also promote the angiogenesis of ECs by DUSP8/ERK1/2 signaling pathway in vivo.

## 4. Discussion

This was the first time to reveal that CSFP stress-modulated OBs to ECs communication via exosomal miRNAs, which was related to the angiogenesis of ECs. First, we made sure that exosomes derived from OBs can be delivered into bEnd.3 cells and CSFP-Exos demonstrated a strong ability to increase the angiogenesis of ECs. Second, we found that the stimulation of CSFP stress could lead OBs to secrete exosomal miR-423-5p to facilitate migration, proliferation, and tube formation of ECs. Finally, we proved that the effect of exosomal miR-423-5p on ECs was realized by targeting DUSP8 and regulating ERK1/2 signaling pathway. All the data indicated that exosomal miR-423-5p originating from CSFP stress-stimulated OBs could promote the angiogenesis of ECs via DUSP8/ERK1/2 signaling pathway.

Exosomes, common membrane-bound nanovesicles that are actively secreted by exocytosis, contain diverse biomolecules, such as proteins, mRNAs, and miRNA [19, 20], which can mediate plenty of physiological and pathological processes in the recipient cells [21–23]. Exosome secretion is a constitutive phenomenon, and both physiological and pathological processes can influence the exosomal surface molecules and the contents [24]. Given our previous studies have already confirmed that CSFP, a special mechanical stimulation, could affect the angiogenesis and osteogenesis of TEL [9, 10, 25], we speculated that CSFP may have an effect on exosomes secretion that mediated cell communication between OBs and ECs.

Bone is a highly vascularized tissue, and mechanical stimulations regulate bone vascularization in the bone microenvironment through modulating all kinds of angiogenic mediators, which is essential for bone formation [26–28]. Here, we found exosomal miR-423-5p acted as a mediator for OBs stimulated by CSFP to promote the angiogenetic capacity of ECs. In glioblastomas, miR-423-5p acted as an angiogenic promoter that contributed to the malignant phenotype by activating the AKT and ERK1/2 signaling pathways [29]. What is more, hADSC-derived exosomal miR-423-5p was a critical miRNA for proangiogenic activity via targeting Sufu [30]. These evidences indicated that miR-423-5p could affect the angiogenesis of ECs. Combined with our results of miRNA-seq, miR-423-5p mimic, miR-423-5p NC, and miR-423-5p inhibitor were used to investigate whether the miR-423-5p played a major role in CSFP-Exos facilitating the angiogenesis of ECs, and our later results proved that the angiogenetic capacity of ECs was significantly increased when miR-423-5p in CSFP-Exos was overexpressed.

DUSP8, a member of the DUSPs family, has been shown to be a physiological inhibitor of MAPK signaling and could regulate cell oxidative stress response, cell apoptosis, and the occurrence and development of diseases [31, 32]. In the metabolic homeostasis or cardiovascular system, DUSP8 could regulate the resistance to diet-induced obesity or cardiac ventricular remodeling by altering ERK1/2 signaling pathway. Here, the result demonstrated that miR-423-5p could inhibit the expression of DUSP8, thereby enhancing the angiogenesis of ECs. To further reveal the specific mechanism, biomarkers in ERK1/2 signaling pathway were detected. Data showed that the activity of ERK1/2 signaling pathway would be improved when miR-423-5p was overexpressed, while DUSP8 was inhibited. In addition, the inhibition of the ERK1/2 signaling pathway could obviously weaken the angiogenesis-promoting effect of miR-423-5p, suggesting the importance of ERK1/2 signaling pathway in promoting angiogenesis.

**Figure 7:**
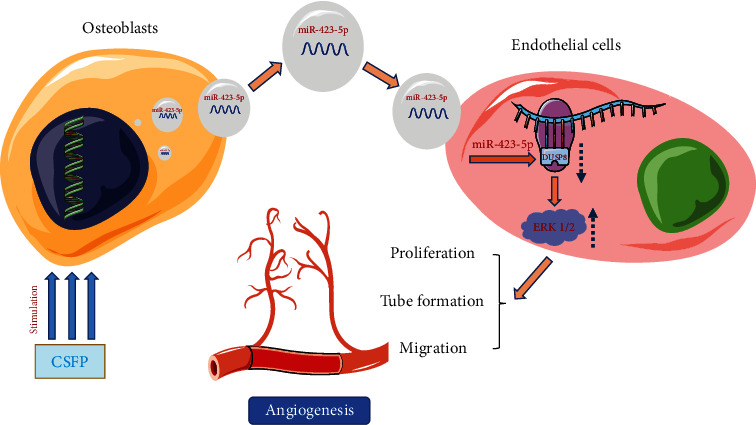
Model of exosomal miR-423-5p derived from CSFP-induced OBs in promoting angiogenesis of ECs. CSFP induces the secretion of OBs-derived exosomal miR-423-5p. OBs-derived exosomal miR-423-5p can directly target DUSP8 and then regulate the ERK1/2 signaling pathway to promote the angiogenesis of ECs.

However, the study has several limitations. Angiogenesis can be regulated by multi kinds of factors, such as cytokines and chemokines [33], and exosomes carry various biomolecules, including protein, lipids, and nucleic acid. Hence, further studies are still needed to reveal the probable exosomal contents derived from OBs that can enhance angiogenesis. Besides, we haven't knocked down DUSP8 to further verify its effect on regulating angiogenesis and ERK1/2 signaling pathway. Despite the above shortcomings, our study clearly indicated that exosomal miR-423-5p derived from CSFP stress-stimulated OBs could enhance the angiogenetic ability of ECs via targeting DUSP8 and then activating ERK1/2 signaling pathway (Figure 7).

## 5. Conclusion

In conclusion, our study was the first time to reveal that exosomes derived from CSFP stress-stimulated OBs could promote the angiogenesis of ECs, and CSFP-Exos may exert facilitating vascular regeneration by transferring miR-423-5p to downregulate DUSP8 and activate ERK1/2 signaling pathway. These findings highlight the effect of exosomes derived from CSFP stress-stimulated OBs on facilitating the angiogenesis of ECs, which enriched our understanding of the interaction between angiogenesis and osteogenesis.

## Figures and Tables

**Table 1:** List of the primer sequences.

Name	Primer sequences
mmu-miR-423-5p	
Forward	5′-CTCAACTGGTGTCGTGGAGTCGGCAATTCA GTTGAGAAAGTCTC-3′
Reverse	5′-ACACTCCAGCTGGGTGAGGGGCAGAGAGC GA-3′
U6	
Forward	5′- CTCGCTTCGGCAGCACA-3′
Reverse	5′-AACGCTTCACGAATTTGCGT-3′

## Data Availability

All data supporting the study are available from the corresponding author upon reasonable request.
